# Failure of chronic hydroxychloroquine in preventing severe complications of COVID-19 in patients with rheumatic diseases

**DOI:** 10.1093/rap/rkab014

**Published:** 2021-03-02

**Authors:** Khanh Pham, Heidi Torres, Michael J Satlin, Parag Goyal, Roy M Gulick

**Affiliations:** 1 NewYork-Presbyterian Hospital, Weill Cornell Medical Center; 2 Department of Medicine, Weill Cornell Medicine, New York, NY, USA

**Keywords:** hydroxychloroquine, rheumatologic diseases, lupus, rheumatoid arthritis, COVID-19, pre-exposure prophylaxis, preventive

## Abstract

**Objective:**

To compare baseline characteristics, clinical presentations and outcomes of patients with rheumatic conditions requiring hospitalization for coronavirus disease 2019 (COVID-19) who received chronic HCQ with those who did not receive chronic HCQ.

**Methods:**

We identified all patients with a rheumatologic disease who were admitted with COVID-19 to two hospitals in New York City between 3 March 3 and 30 April 2020. Patients who received chronic HCQ prior to admission were matched 1:2 (±10 years of age) with patients who did not receive chronic HCQ. We compared demographics, comorbidities, HCQ dosages, concurrent medications, presentations and outcomes between the groups.

**Results:**

There were 14 patients receiving HCQ and 28 matched control subjects. The median age of cases was 63 years [interquartile range (IQR) 43–73) and 60 years (IQR 41–75) for controls. Control subjects had a higher prevalence of pulmonary diseases (42.8%), diabetes (35.7%) and obesity (35.7%) than their case counterparts (28.6%, 14.3% and 7.1%, respectively). A higher proportion of cases than control subjects (50% *vs* 25%) reported the use of prednisone for their rheumatic conditions prior to admission. Despite these differences in baseline characteristics, univariate logistic regression revealed no statistically significant differences in the need for mechanical ventilation [OR 1.5 (95% CI 0.34, 6.38)] or in-hospital mortality [OR 0.77 (95% CI 0.13, 4.56)].

**Conclusion:**

HCQ therapy in individuals with rheumatic conditions was not associated with less severe presentations of COVID-19 among hospitalized patients compared with individuals with rheumatic conditions not receiving HCQ.

Rheumatology key message

- Chronic hydroxychloroquine therapy in patients with rheumatic conditions did not prevent severe COVID-19.

## Introduction

Coronavirus disease 2019 (COVID-19) is a highly transmissible disease caused by severe acute respiratory syndrome coronavirus 2 (SARS-CoV-2) that was first detected in Wuhan, China in December 2019 [1] and has rapidly become a pandemic with >81.5 million cases and >1.7 million deaths worldwide as of late December 2020 [2]. SARS-CoV-2 often causes a viral pneumonia with frequent complications, including hypoxic respiratory failure, acute respiratory distress syndrome, coagulation dysfunction, cytokine storm syndrome, multiorgan failure and death [3, 4]. Several drugs have been investigated to treat and prevent this disease. With the exception of vaccines that have recently been granted emergency use authorization (EUA) status by the US Food and Drug Administration (FDA), there is no other effective preventive agent for COVID-19 [5].

HCQ is FDA approved for malaria and some rheumatic diseases, but also demonstrates *in vitro* activity against SARS-CoV-2 [6, 7]. Since the onset of the pandemic, however, numerous randomized controlled trials (RCTs) have found that HCQ is unlikely to be beneficial as a therapeutic agent when used in hospitalized patients with COVID-19 [8–13]. One study even reported that among patients who did not undergo mechanical ventilation at baseline, those in the HCQ group, as compared with the usual care group, had a higher frequency of invasive mechanical ventilation or death [13].

There are studies that have also assessed the preventive effects of HCQ in COVID-19. One RCT that evaluated the efficacy and safety of HCQ for pre-exposure SARS-CoV-2 prophylaxis among healthcare workers found that daily HCQ did not prevent infection, although this study was terminated early and did not reach enrolment targets [14]. In particular, the efficacy of HCQ in the rheumatic patient population has only been reported in a small number of studies [15–20], with most demonstrating no benefit of HCQ in preventing hospital admission with COVID-19, except for one study that reported a lower risk of COVID-19 in patients taking HCQ as compared with patients taking DMARDs [20]. In our study, we compared the baseline characteristics, clinical presentations and outcomes of patients with rheumatic conditions requiring hospitalization for COVID-19 who received chronic HCQ with those who did not receive chronic HCQ.

## Methods

We reviewed the medical records of all patients who presented to NewYork-Presbyterian Hospital/Weill Cornell Medical Center (quaternary referral center) and the affiliated Lower Manhattan Hospital (community hospital) from 3 March to 30 April 2020 with confirmed COVID-19, defined as having SARS-CoV-2 nucleic acid detected by reverse transcription polymerase chain reaction (RT-PCR) using a nasopharyngeal swab sample. We identified the proportion of these patients who were hospitalized and had underlying rheumatic diseases and divided them into individuals who were receiving chronic HCQ (cases) and those not receiving the drug (controls). A random number generator was used to individually match two eligible control patients to each eligible case patient within the same 10 year age category, thereby achieving a 2:1 matching by age (±10 years). We then reviewed the electronic medical record to report on the demographics, underlying comorbidities, HCQ dosages, concurrent medications, presentations and outcomes of these two groups. All patients were followed until hospital discharge. We applied descriptive statistics, using medians for continuous variables and proportions for categorical variables, and used univariate logistic regression to analyze the outcomes. Stata 16.1 software (StataCorp, College Station, TX, USA) was used for all analyses. This study was approved by the Institutional Review Board at Weill Cornell Medicine with a waiver of informed consent.

## Results

Table 1 summarizes the baseline characteristics and presenting features on hospital arrival of the case and control patients. Of the 1863 patients who presented to our hospitals with COVID-19 between 3 March and 30 April 2020, we identified 14 case subjects with rheumatic diseases who were receiving chronic HCQ before hospital admission and 28 matched control subjects with rheumatic diseases who were not receiving chronic HCQ. The median age for case subjects was 63 years [interquartile range (IQR) 43–73] and 60 years (IQR 41–75) for control subjects. Both groups were diverse racially and ethnically, although there was a greater representation of Black individuals (28.6% *vs* 10.7%) and Hispanic ethnicity (28.6% *vs* 17.9%) in cases as compared with controls. Women accounted for 85.7% of cases and 67.9% of controls. Of the case subjects, 7 (50%) had RA, 5 (35.7%) had SLE and 3 (21.4%) reported other rheumatic conditions. By comparison, 11 (39.3%) control subjects had RA, 2 (7.1%) had SLE and 16 (57.1%) reported other rheumatic conditions, which included psoriasis, mixed connective tissue disease, vasculitides and other autoimmune conditions. Case individuals were receiving 200 or 400 mg/day of HCQ for at least 4 weeks prior to presentation. A significant proportion of case and control individuals reported taking other concurrent immunomodulating agents prior to admission. Notably, a larger proportion of case subjects (50%) than controls (25%) received prednisone prior to presentation.

**Table 1:** Baseline characteristics and presenting features of patients with rheumatic diseases and COVID-19 diagnosis who were taking HCQ and control patients

Characteristics	Taking HCQ (*n* = 14)	Control patients (*n* = 28)
Age, years, median (IQR)	63 [43–73]	60 [41–75]
Female, *n* (%)	12 (85.7)	19 (67.9)
Race, *n* (%)		
White	4 (28.6)	10 (35.7)
Black	4 (28.6)	3 (10.7)
Asian	2 (14.3)	7 (25)
Other	2 (14.3)	5 (17.9)
Not specified	2 (14.3)	3 (10.7)
Hispanic ethnicity, *n* (%)	4 (28.6)	5 (17.9)
Rheumatic diagnosis, *n* (%)		
RA	7 (50)	11 (39.3)
SLE	5 (35.7)	2 (7.1)
Other	3 (21.4)	16 (57.1)
Comorbid conditions, *n* (%)		
Hypertension	11 (78.6)	18 (64.3)
Coronary artery disease	5 (35.7)	5 (17.9)
Diabetes	2 (14.3)	10 (35.7)
End-stage renal disease	2 (14.3)	3 (10.7)
Obesity (BMI ≥30)	1 (7.1)	10 (35.7)
Congestive heart failure^a^	0	3 (10.7)
Pulmonary disease, *n* (%)	4 (28.6)	12 (42.8)
COPD	2 (14.3)	2 (7.1)
Asthma	1 (7.1)	6 (21.4)
Interstitial lung disease	1 (7.1)	1 (3.6)
Other	0	3 (10.7)
HIV, *n* (%)	0	1 (3.6)
Active malignancy, *n* (%)	0	0
Solid organ transplant recipient, *n* (%)	2 (14.3)	1 (3.6)
Taking immunomodulators, *n* (%)		
Prednisone <20 mg/day	6 (42.9)	7 (25)
Prednisone ≥20 mg/day	1 (7.1)	0
Mycophenolate	5 (35.7)	1 (3.6)
Tacrolimus	4 (28.6)	1 (3.6)
Methotrexate	4 (28.6)	4 (14.3)
TNF-α inhibitor	0	4 (14.3)
Other monoclonal antibody	1 (7.1)	3 (10.7)
Other immunomodulator	0	4 (14.3)
Presenting symptoms, *n* (%)		
Dyspnea	12 (85.7)	22 (78.6)
Fever	10 (71.4)	21 (75)
Cough	10 (71.4)	19 (67.9)
Diarrhea	6 (42.9)	11 (39.3)
Nausea or vomiting	5 (35.7)	8 (28.6)
Myalgias	3 (21.4)	2 (7.1)
Chest pain	2 (14.3)	8 (28.6)
Abdominal pain	1 (7.1)	0
Headache	0	5 (17.9)
Sore throat	0	5 (17.9)
Altered mental status	0	3 (10.7)
Ageusia (loss of taste)	0	3 (10.7)
Rhinorrhea	0	2 (7.1)
Anosmia (loss of smell)	0	1 (3.6)
Initial chest X-ray findings, *n* (%)		
Infiltrates	12 (85.7)	19 (67.9)
Unilateral infiltrates	0	4 (14.3)
Pleural effusion or other	1 (7.1)	0
No infiltrate or effusion	2 (14.3)	5 (17.9)
Oxygen support within first 3 h of hospital arrival, *n* (%)	6 (42.9%)	17 (60.7%)
Cannula	4 (28.6)	13 (46.4)
Non-rebreather	0	3 (10.7)
Mechanical ventilation	2 (14.3)	1 (3.6)
QTc interval on initial electrocardiogram, median (IQR)	449 (434–474)	428 (412–456)

COPD, chronic obstructive pulmonary disease; QTc interval, rate-corrected QT interval using Bazett’s formula.

a Heart failure with preserved ejection fraction or heart failure with reduced ejection fraction <50%.

Common comorbid illnesses included hypertension, coronary artery disease, diabetes, end-stage renal disease, obesity, congestive heart failure and various pulmonary diseases in both groups. Control subjects had a higher prevalence of diabetes (35.7%) and obesity (35.7%) than their case counterparts (14.3% and 7.1%, respectively). Additionally, a larger proportion of control subjects (42.8%) than cases (28.6%) had a history of pulmonary disease, which included chronic obstructive pulmonary disease, asthma, interstitial lung disease and other pulmonary conditions.

Common presenting symptoms between the two groups were dyspnea, fever, cough, diarrhea and nausea or vomiting. Controls were more likely to have chest pain, headache, sore throat, altered mental status, ageusia, rhinorrhea and anosmia than case subjects. The majority of individuals in both groups had radiographic findings of bilateral pulmonary opacities consistent with viral pneumonia. Of the 14 case individuals, 6 (42.9%) required oxygen support within the first 3 hours of hospital arrival, as compared with 17 of 28 (60.7%) control individuals. Among those taking chronic HCQ, the median baseline QTc interval on the initial electrocardiogram was 449 msec (IQR 434–474), as compared with a median baseline QTc of 428 msec (IQR 412–456) among those not taking chronic HCQ.

Table 2 summarizes the hospital course and outcomes of the case and control subjects. Common complications between the two groups included acute kidney injury, acute respiratory distress syndrome, need for renal replacement, venous thromboembolism event, coinfection and other complications. Notably, cases experienced nearly more than twice the rate of acute kidney injury (64.3% *vs* 32.1%), bacterial coinfection (14.3% *vs* 7.1%), septic shock (21.4% *vs* 10.7%) and myocardial infarction (14.3% *vs* 7.1%) than controls. Two (14.3%) case patients also developed deep vein thrombosis, while none of the control patients developed this complication. Despite differences in the proportion of individuals in each group experiencing these complications, there were no statistically significant differences noted when univariate logistic regression was applied.

**Table 2:** Hospital course and final outcomes of patients with rheumatic diseases taking HCQ *vs* control patients

Event	Taking HCQ^a^ (*n* = 14)	Control patients (*n* = 28)	Univariate logistic regression	
			Odds ratio (95% CI)	*P*-value
Complications, *n* (%)				
Acute kidney injury	9 (64.3)	9 (32.1)	3.8 (0.98, 14.67)	0.053
ARDS	4 (28.6)	7 (25)	1.2 (0.28, 5.07)	0.80
Need for renal replacement	1 (7.1)	3 (10.7)	0.64 (0.06, 6.79)	0.71
VTE event				
Deep vein thrombosis	2 (14.3)	0	1.0^c^	–^c^
Pulmonary embolism	0	1 (3.6)	1.0^c^	–^c^
Coinfection				
Bacterial	2 (14.3%)	2 (7.1%)	2.2 (0.27, 17.27)	0.47
Fungal	1 (7.1%)	0	1.0^c^	–^c^
Viral	0	1 (3.6)	1.0^c^	–^c^
Unspecified	0	1 (3.6)	1.0^c^	–^c^
Other complications, *n* (%)				
Septic shock	3 (21.4)	3 (10.7)	2.3 (0.39, 13.08)	0.36
VAP	3 (21.4)	2 (7.1)	3.5 (0.52, 24.26)	0.19
Arrhythmia	2 (14.3)	3 (10.7)	1.4 (0.20, 9.45)	0.74
Myocardial infarction	2 (14.3)	2 (7.1)	2.2 (0.27, 17.27)	0.47
CHF or cardiogenic shock	0	2 (7.1)	1.0^c^	–^c^
Antiviral or immunomodulator^b^, *n* (%)				
HCQ	14 (100)	17 (60.7)	1.0^c^	–^c^
Corticosteroids	10 (71.4)	13 (46.4)	2.9 (0.73, 11.43)	0.13
Remdesivir	1 (7.1)	1 (3.6)	2.1 (0.12, 35.89)	0.62
Sarilumab	0	1 (3.6)	1.0^c^	–^c^
Mechanical ventilation at any point, *n* (%)	4 (28.6)	6 (21.4)	1.5 (0.34, 6.38)	0.61
Extubated	2 (14.3)	3 (10.7)	1.0 (0.079, 12.56)	1.00
New tracheostomy	2 (14.3)	3 (10.7)	2.0 (0.11, 35.81)	0.64
ICU admission at any point, *n* (%)	4 (28.6)	6 (21.4)	1.5 (0.34, 6.38)	0.61
Discharged from ICU	3 (75)	4 (67)	1.5 (0.089, 25.39)	0.78
Death, *n* (%)	2 (14.3)	5 (17.9)	0.77 (0.13, 4.56)	0.77
Discharged from hospital, *n* (%)	12 (85.7)	23 (82.2)	1.0^c^	–^c^
Home	9 (64.3)	19 (67.9)	0.85 (0.22, 3.29)	0.82
Subacute rehab	1 (7.1)	2 (7.1)	1.0 (0.083, 12.07)	1.00
Acute rehab	2 (14.3)	1 (3.6)	4.5 (0.37, 54.54)	0.24
Skilled nursing facility	0	1 (3.6)	1.0^c^	–^c^

ARDS: acute respiratory distress syndrome; VTE: venous thromboembolism; VAP: ventilator-associated pneumonia; CHF: congestive heart failure.

a Prior to hospital admission.

b Received during hospital course.

c No CI or *P*-value calculated.

More case individuals as compared with control individuals received HCQ (100% *vs* 60.7%), corticosteroids (71.4% *vs* 46.4%) and remdesivir (7.1% *vs* 3.6%) during the course of their hospitalization.

Regarding the rest of the hospital course, there were similar rates of intubation and intensive care unit (ICU) admission between the cases and controls (28.6% and 21.4%, respectively). Of the 4 case patients requiring ICU admission, 3 (75%) were eventually discharged from the ICU. In comparison, 4 (67%) of the 6 control patients were in stable condition to eventually leave the ICU. Overall, 14.3% of case patients died during their hospitalization, as compared with 17.9% of control patients. When analyzed by univariate logistic regression, there were no statistical differences noted in the need for mechanical ventilation [OR 1.5 (95% CI 0.34, 6.38)] or in-hospital mortality [OR 0.77 (95% CI 0.13, 4.56)] between the two groups.

## Discussion

Thus far in the COVID-19 pandemic, vaccines are the only proven agents to prevent COVID-19 [5]. While numerous randomized clinical trials have found that HCQ is ineffective as a therapeutic and preventive agent [8–14], fewer have studied the effects of this agent as pre-exposure prophylaxis in the rheumatic patient population [15–20]. Our study is one of a small number of studies to show that the use of chronic HCQ for a variety of rheumatic diseases failed to prevent COVID-19 [15–20].

Our study illustrates several important findings. First, our patients were racially and ethnically diverse and nearly all had comorbid illnesses considered to be risk factors for developing severe COVID-19, such as diabetes, hypertension, obesity and chronic heart, lung and kidney diseases [3, 4]. In addition, a significant proportion of individuals in each group were receiving other concurrent immunomodulating agents prior to admission that may also increase the risk of acquiring and developing severe COVID-19 [19]. It should also be noted that the prevalence of diabetes, obesity and pulmonary diseases was higher in the control than case patients. Additionally, a much higher proportion of case individuals, as compared with control individuals, received corticosteroids before and during their hospitalization. Despite these baseline differences, there were no statistical differences observed in the outcomes between these groups, which included no differences in complication rates, need for mechanical ventilation or ICU care, in-hospital mortality or hospital discharge. Although there were no statistical differences observed in hospital outcomes, our findings may help clinicians to identify vulnerable patients who may be at risk for developing severe COVID-19 illness.

Our study has several limitations. First, our sample size was small, which limited our ability to detect potential differences between groups and adjust for potential confounders, and evaluated patients from only two hospitals. Second, adherence to HCQ among case patients prior to admission was not examined—the individual HCQ regimen and history were obtained from chart review, which did not assess medication adherence.

In summary, our study suggests that chronic HCQ use for rheumatic diseases did not prevent the development of severe COVID-19 when compared with a control group that was not receiving chronic HCQ. These data, combined with other data that have not identified a protective effect of HCQ, inform clinicians that patients with rheumatic diseases who are taking long-term HCQ should be monitored closely for severe illness if they develop COVID-19.

## Data Availability

The data underlying this article will be shared on reasonable request to the corresponding author.
